# Evaluation of a portable retinal imaging device: towards a comparative quantitative analysis for morphological measurements of retinal blood vessels

**DOI:** 10.1098/rsos.230065

**Published:** 2023-06-21

**Authors:** M. Elena Martinez-Perez, Alun D. Hughes, Simon A. McG. Thom, Kim H. Parker, Nicholas W. Witt

**Affiliations:** ^1^ Department of Computer Science, Institute of Research on Applied Mathematics and Systems, National Autonomous University of Mexico, Mexico City, Mexico; ^2^ MRC Unit for Lifelong Health and Ageing, Institute of Cardiovascular Science, University College London, Gower Street, London WC1E 6BT, UK; ^3^ National Heart and Lung Institute, Imperial College London, Hammersmith Campus, London W12 0HS, UK; ^4^ Department of Bioengineering, Imperial College, London, South Kensington Campus, London SW7 2AZ, UK

**Keywords:** retinal image devices, portable imaging, quantitative analysis, fundus images

## Abstract

This study investigated the possibility of using low-cost, handheld, retinal imaging devices for the automatic extraction of quantifiable measures of retinal blood vessels. Initially, the available handheld devices were compared using a Zeiss model eye incorporating a USAF resolution test chart to assess their optical properties. The only suitable camera of the five evaluated was the Horus DEC 200. This device was then subjected to a detailed evaluation in which images in human eyes taken from the handheld camera were compared in a quantitative analysis with those of the same eye from a Canon CR-DGi retinal desktop camera. We found that the Horus DEC 200 exhibited shortcomings in capturing images of human eyes by comparison with the Canon. More images were rejected as being unevaluable or suffering failures in automatic segmentation than with the Canon, and even after exclusion of affected images, the Horus yielded lower measurements of vessel density than the Canon. A number of issues affecting handheld cameras in general and some features of the Horus in particular have been identified that might contribute to the observed differences in performance. Some potential mitigations are discussed which might yield improvements in performance, thus potentially facilitating use of handheld retinal imaging devices for quantitative retinal microvascular measurements.

## 1. Introduction

Examination of the retinal fundus has been practised since the nineteenth century [1]. Retinopathy and other qualitative manifestations of retinal microvascular disease predict future cardiovascular risk even in individuals without diabetes or hypertension [2]. More recently, subtle alterations in quantitative measures of the retinal vasculature (e.g. arterial/venous ratios, fractal dimension (FD) and the optimality of arterial bifurcations) have been shown to predict subsequent cardiovascular disease, as well as progression of diabetes [3,4]. Such quantitative measurements can identify individuals at increased cardiovascular risk in epidemiological studies before such disease is evident as retinopathy or from other clinical observations [5,6].

Quantitative analysis of the retinal vasculature is typically performed on images captured by a desktop fundus camera located in a clinic. However, these facilities are not necessarily accessible by patients with impaired mobility or in remote or rural locations and may only rarely be available in low-income countries. Accordingly, it would be desirable to be able to capture images for quantitative analysis from portable devices in a community setting.

A growing number of portable retinal imaging devices are on the market, frequently (but not exclusively) in the form of an add-on to a smartphone. Evaluation of several such devices has been reported in the setting of qualitative assessment of diabetic retinopathy and glaucoma [7,8], increasingly coupled with artificial intelligence approaches to classification [9]. However, the imaging requirements for the quantitative assessment of the retinal microvasculature are more demanding than for qualitative assessment of retinopathy, and it remains uncertain whether portable retinal imaging devices are suitable for this purpose.

The objective of this work was therefore to explore the feasibility of making quantitative measurements of geometrical and morphological features of the retinal vasculature in images taken with portable commercial devices. For this purpose, five commercial portable retinal imaging devices were identified, as available in UK at the time of initiation of our study. These devices offered a diverse range of capabilities; four were smartphone-based, whereas one was self-contained and incorporated functionality closer to that of a conventional fundus camera. Accordingly, an initial assessment of all five portable available devices was performed to objectively compare their capability and consider to what extent they may be suitable to support quantitative measurements of the retinal vasculature.

## 2. Methods

### 2.1. Initial assessment of available devices

The five available devices considered were:

— D-EYE (D-EYE, Padova, Italy) [10] (evaluated with iPhone 6S)—uses the LED light source of the smartphone. A dedicated smartphone app controls the illumination and image capture.
— Peek Retina (Nesta, London, UK) [11] (evaluated with iPhone 6S)—provides its own battery-powered light source. Images are captured using the native camera app of the smartphone.
— Welch Alleyn Panoptic Ophthalmoscope (Welch Allyn, Skaneateles Falls, NY) [12] (evaluated with iPhone 6S)—provides its own battery-powered light source. A dedicated smartphone app facilitates capture and storage of images, but images can also be captured using the native camera app of the smartphone.
— Volk iNview (Volk Optical, Mentor, OH, USA) [13] (evaluated with 6th Gen iPod)—uses the LED light source of the iPod. A dedicated app controls the illumination and image capture.
  (the four above are designed to operate in conjunction with the Apple iPhone and/or iPod).
— Horus DEC 200 (MiiS, Hsinchu, Taiwan) [14]—employs its own built-in camera, using infrared illumination for image composition and white light flash for image capture.

Taking into account the diverse capabilities of the different devices, we performed an objective comparison of their basic optical performance based on analysis of images captured by each device of an adapted Zeiss model eye (Carl Zeiss, Inc.). The Zeiss model eye emulates a fully dilated pupil. It has been adapted to accommodate a USAF resolution test chart (Edmund Optics, USA) [15] consisting of Groups 2 and 3, at the position of the macula, thus allowing the optical performance of the various devices to be characterized.

A Canon CR-DGi non-mydriatic retinal fundus camera (Canon Inc) [16] equipped with an EOS-10D SLR back was also included for comparison. This is typical of desktop devices commonly used to capture retinal images for quantitative analysis and it is considered in the present context to represent the gold-standard of retinal imaging performance.

The initial suitability of portable available devices was assessed against the following criteria:

— Requirement for mydriasis—in a community setting, capture of images without administration of mydriatic agents is highly desirable.
— Field of view (FOV)—measurements of length/diameter ratio of vascular segments or optimality of individual bifurcations exhibit considerable scatter, and an adequate FOV is necessary to yield sufficient measurements to give a reliable measure of central tendency. Typically, published studies on measurement of retinal vascular geometry employ images with a FOV in the range 30° to 45° [17]. A smaller FOV would compromise the utility of the image for this purpose.
— Optical performance—this is represented firstly by the number of pixels per degree of FOV of the device, taking into account that the retinal image does not in all cases occupy the full field of the sensor. This can be considered as a figure of merit indicating the likely precision that could be achieved in measurements of vascular calibre. Furthermore, the optical resolution (lines mm^−1^) assessed from the USAF test chart installed in the model eye additionally takes into account the optical performance of the device.

Each portable imaging device was handheld so as to represent normal operation, and manually positioned in front of the model eye so as to optimize the composition of the image in accordance with the manufacturer’s guidance. Images from each device were captured for subsequent analysis wherever possible from the native imaging device, and otherwise from the smartphone application provided by the manufacturer. The operator, who was an experienced retinal photographer, underwent extensive training in the use of the handheld devices prior to initiation of the study.

**Figure 1.**
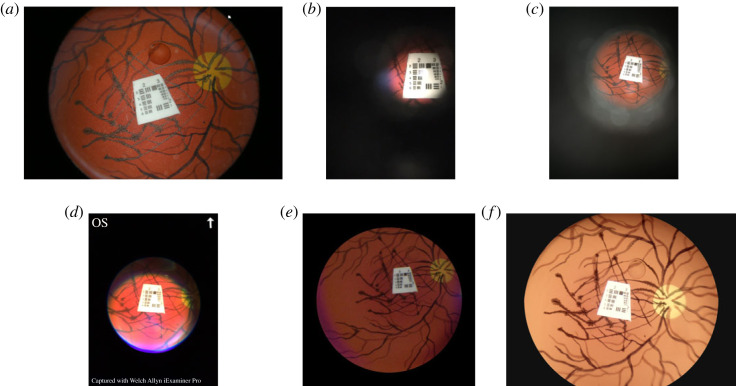
Photographs taken of the Zeiss model eye with the USAF resolution test chart. (*a*) Canon CR-DGi non-mydriatic desktop fundus camera, (*b*) D-Eye, (*c*) Peek Retina, (*d*) Welch Alleyn, (*e*) Volk iNview and (*f*) Horus DEC 200.

The images from each device were assessed visually to establish the highest resolution at which the lines of the appropriate Group/Element of the USAF test chart could be separated. Additionally, the FOV was evaluated by comparison with images of the model eye captured by the Canon CR-DGi retinal camera with a fixed 45° field (figure 1). A figure with a zoomed version of the USAF test chart can be found in the electronic supplementary material.

An assessment was also performed of the feasibility of capturing images without mydriasis in a healthy volunteer, after sitting in a darkened room for 5 min.

### 2.2. Evaluation of Horus DEC 200 in healthy volunteers

The evaluation was performed with healthy volunteers, and sought to compare images of the same subject captured by the Horus portable device (figure 1*f*) with those from the Canon CR-DGi desktop camera (our gold standard, figure 1*a*). A total of 46 subjects were recruited from staff and students at University College London (UCL). Ethical approval was given by the UCL Research Ethics Committee (Project ID no. 6782/001) and written informed consent was obtained from all participants. The ethics approval allowed for recruitment of volunteers in the age range 18–70 years old. No exclusion criteria were applied. The median age of the volunteers was 34 years old, and 59% were female. Each volunteer was photographed by both the Horus and Canon retinal cameras in accordance with the following protocol:

— Subjects were randomized using Excel software (Microsoft Inc.) to have photography with the Canon camera followed by the Horus, or vice versa.
— Subjects were placed in a dark room (or both eyes covered) for 5 min prior to photography.
— With each camera, photography commenced in the right eye, followed by the left eye after a delay of at least 2 min.
— In the event that the photographer considered any image to be unevaluable, one further attempt was permitted after a delay of at least 2 min.

Following completion of photography, a qualitative assessment of evaluability of each image was performed to indicate the number of subjects and images expected to be capable of meaningful quantitative comparison between the two cameras. Qualitative assessment was performed by subjective visual inspection of the images displayed on a computer screen. A grid, positioned with respect to the optic disc (OD), was superimposed on the image to guide the assessment. At its furthest point, the grid extended to 6.5 × the disc radius from the disc centre towards the macula but was truncated at the vertical extremes to provide a margin between the grid and the edge of the image. Assessment was performed on two quadrants per image, and each quadrant was considered to be evaluable if free of serious artefacts that would impede measurements, and the major vascular trees were visualized to the boundary of the grid. For the purposes of the unsupervised automatic assessment, an image was judged acceptable provided at least one quadrant was evaluable. From this assessment, pairs of images (one from each camera) of the same eye were identified to go forward for quantitative comparison.

**Figure 2.**
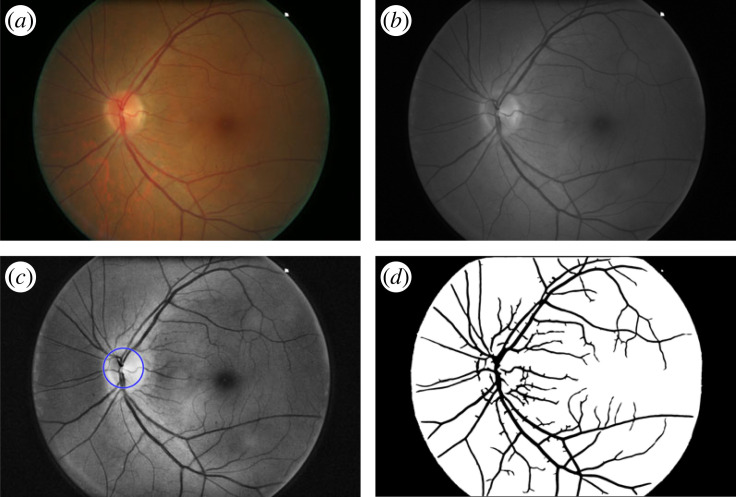
(*a*) Original Canon photograph, (*b*) green channel, (*c*) green channel after adaptive histogram equalization (AHE), with the optic disc (OD) detection marked in blue, (*d*) segmented blood vessels in black.

Post-processing of the images was performed. The green channel (figure 2*b*) was extracted from the colour image (figure 2*a*) to facilitate detection of the blood vessels. Then an adaptive histogram equalization (AHE) was applied to the green channel to improve the contrast in the images, which was sometimes inhomogeneous due to poor lighting. AHE differs from ordinary histogram equalization in that the adaptive method computes several histograms, each corresponding to a distinct section of the image, and uses them to redistribute the lightness values of the image. It is therefore suitable for improving the local contrast and enhancing the definitions of edges in each region of an image [18]. Figure 2*b*,*c* shows the green channel before and after the AHE, respectively. The segmentation of blood vessels was then performed to separate the retinal vasculature from the background.

Blood vessels were segmented using a previously described algorithm based on multi-scale analysis [19]. Two geometrical features were used based on the first and second spatial derivatives of the intensity image that gives information about vessel topology. The local maxima over scales of the magnitude of the gradient and the maximum principal curvature of the Hessian tensor were used in a multiple pass region growing procedure. The algorithm was tested on red-free and fluorescein retinal images, taken from two local and two public databases. Comparison with public databases yielded an average true positive rate (TPR) of 74% and 4% false positive rate (FPR) (see figure 2*d* blood vessels shown in black).

The area corresponding to the OD was masked in order to establish binary rooted trees. To accomplish this, we applied a previously developed automatic method [20] that: (i) uses the three channels (RGB) of the digital colour image to locate the region of interest (ROI) where the OD lies, (ii) measures the Shannon information content per channel in the ROI, to decide which channel is most appropriate for searching for the OD centre using the circular Hough transform. The performance of this multi-spectral algorithm was evaluated in two public databases yielding a TPR = 88% and FPR = 3% (see figure 2*c* where OD is marked in blue).

**Figure 3.**
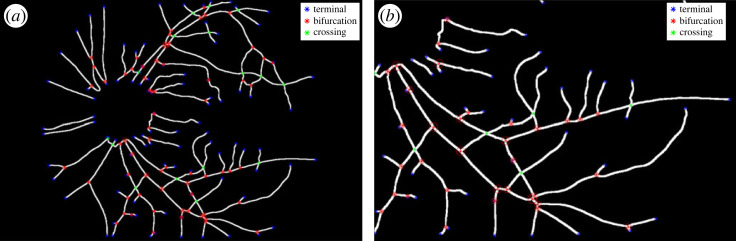
(*a*) Skeleton found by a thinning process with the characterization of edges (vessel segments) and nodes (terminal, branching and crossing points) and (*b*) zoom of (*a*), where terminal points are marked in blue, branching points in red and crossing points in green. All the nodes are obtained automatically, in some cases where a crossing is very close to a bifurcation, the system cannot resolve the crossing automatically and it is kept as a bifurcation.

Following vessel and OD automatic segmentation, the skeletons of the vascular trees were obtained by a thinning process where pixels were eliminated from the boundaries towards the centre without destroying connectivity [21] (see figure 3*a*). Segments of the skeletons are characterized as edges and the terminal, bifurcating and crossing points as nodes in a graph representation (figure 3*b*). The following four metrics were calculated for each image and a pairwise comparison between the two cameras was derived.

1. Area ratio (%) between vessel (black pixels) and background (white pixels) inside the FOV region (figure 2*d*). This is a metric of vessel density.
2. Length of edges between nodes. This metric gives the distribution of vessel lengths in the image.
3. Number of nodes categorized by degree. Degree 1 = terminal point, degree 3 = bifurcation point and degree 4 = crossing point. This metric gives the distribution of bifurcation, crossing and terminal points in the image.
4. FD. This is a measure of the complexity of the vasculature branching pattern. For a two-dimensional image, the value of FD is between 1 (line) and 2 (plane), and it refers to how much of the space is filled by the graph. To compute this value, we used the box counting algorithm which covers the skeletonized graph with a regular grid of mesh size *s*, and counts the number of grid boxes which contain at least some of the structure *N*. *s* is changed to progressively smaller values and the count is repeated to give *N*(*s*). FD is defined
  $${\mathrm{FD}}_{\mathrm{box}}=\lim_{s\to 0}\frac{\log(N(s))}{\log(1/s)};$$
  and it is approximated by computing the slope of the fitted line of the log (*N*(*s*)) versus log (1/*s*) plot [22].

#### 2.2.1. Statistical analysis

All statistical analyses were performed using Stata 17.0 MP-Parallel Edition (StataCorp) or custom-written Matlab functions (Mathworks Inc). Continuous data were summarized as means and standard deviations (s.d.) and categorical data as frequencies and percentages. Evaluability was compared with a two-sample test of proportions, and under- and over-segmentation proportions using a Fisher’s exact test due to the small sample size in some cells and the paired comparison of evaluable versus non-evaluable images for the Canon and Horus devices was performed using an exact McNemar test. For all quantitative metrics, Canon segmented images were compared with those of Horus from the same eye using a Bland & Altman analysis [23], and agreement was quantified as mean difference and limits of agreement (1.96 times the s.d. of the differences). A Bradley & Blackwood (BB) test [24] was used to test the joint hypothesis of equal means and equal variances in the Bland & Altman analysis and a test of the regression of differences on sums was also performed [25]. For all statistical tests, the null hypothesis was rejected if *α* < 0.05.

### 2.3. Assessment of blood vessel segmentation

For the assessment of the four metrics mentioned in §2.2, we carried out two types of evaluation: (i) we used the original 56 automatically segmented image pairs (total of 112 images) without imposing additional quality criteria (‘unsupervised’ images), and (ii) the automatically segmented images were visually inspected and selected using the following criteria: regardless of the camera type used, images that have less than three-quarters of the blood vessels segmented inside the FOV image area are considered as under-segmented, and those with the appearance of lacework-like vessels in elongated patches in the background are characterized as over-segmented. We eliminated the pairs of automatically segmented images with under- or over-segmented characteristics, leaving a total of 33 pairs of segmented images for analysis, which we will refer to as the ‘curated’ images.

## 3. Results

### 3.1. Results from initial assessment of available devices

The only portable device found to be capable of capturing adequate images was the Horus DEC 200. The Welch Alleyn device supports multiple FOV, but while the smaller fields are claimed to be capable of non-mydriatic operation, this was found to yield very poor images and at the widest FOV mydriasis is essential. The Volk iNview is claimed to support non-mydriatic photography by the manufacturer, but this was not found to be feasible.

The results of the assessment, including those derived from images of the Zeiss model eye are summarized in table 1. The ‘image size’ column shows the size in pixels of the image available from the native camera where this was accessible, or otherwise of the image returned by the corresponding dedicated smartphone application. However, since the retinal field does not in all cases extend to the edge of the image, the pixels per degree of FOV provides a better means of comparison between devices.

**Table 1.** Comparative evaluation of portable retinal imaging available devices.

device	requires mydriasis	∼FOV (${\;}^{\circ }$)	image size (pixels)	∼pixels/FOV	optical resolution (lines mm^−1^)
Canon CR-DGi (+EOS 10D)	no	45	3072 × 2048	58	>14.2
D-EYE	yes	12	2016 × 1512	51	12.7
Peek Retina	yes	23	4032 × 3024	53	14.2
Welch Alleyn	yes	23	4032 × 3024	65	12.7
Volk iNview	yes	50	930 × 930^a^	13^a^	6.3
Horus DEC 200	no	45	2560 × 1920	40	11.3

^a^Evaluated using 6th Gen iPod; 24% improvement available with iPhone 6S.

The D-EYE (figure 1*b*), Peek Retina (figure 1*c*) and Welch Alleyn (figure 1*d*) devices all offer creditable optical performance, but suffer from a smaller FOV than desirable for the measurement of vascular geometry. The FOV of the Welch Alleyn device appears to be limited by the field of illumination rather than the imaging optics. Furthermore, none of these available devices were found to be capable of yielding adequate visualization of the vascular trees without mydriasis. The reader should be reminded that the images shown in figure 1, are taken of the model eye which emulates a fully dilated pupil. Accordingly, they were not considered appropriate for more detailed evaluation.

The Volk iNview (figure 1*e*) has a large FOV which would be suitable for measurement of vascular geometry, although it does require mydriasis. However, this device offers only a very small number of pixels per degree FOV and poor optical resolution (table 1). Both these features will limit the quality of measurements of vessel calibre. The manufacturer advised us that the small number of pixels was a fundamental limitation of the hardware. This was confirmed by our own investigations; the retinal field was focused only on a small region of the smartphone sensor. In this context, it should be noted that the Volk iNview images in this assessment were recorded on a 6th generation iPod, which has a smaller number of pixels than the iPhone 6S used for other devices (3264 × 2448 compared with 4032 × 3024, respectively), since the appropriate adaptor for the iPhone was not available to us at the time. Accordingly, we estimate that an approximate 24% increase in the number of pixels per degree FOV might be obtained by using an iPhone 6S instead of the iPod (assuming an equivalent FOV in both native cameras), but this would still not yield results from the Volk iNview comparable with other devices.

The Horus DEC 200 (figure 1*f*) can capture images without mydriasis and offers a FOV that is appropriate for measurement of vascular geometry. The number of pixels per degree FOV is modest compared with the Canon fundus camera, but it is credible that vascular calibre could be measured with acceptable quality [26]. Of all the portable available devices assessed against the prespecified criteria, the Horus DEC 200 was the only device appropriate for further assessment in healthy volunteers.

### 3.2. Results from qualitative assessment of the Horus images

**Figure 4.**
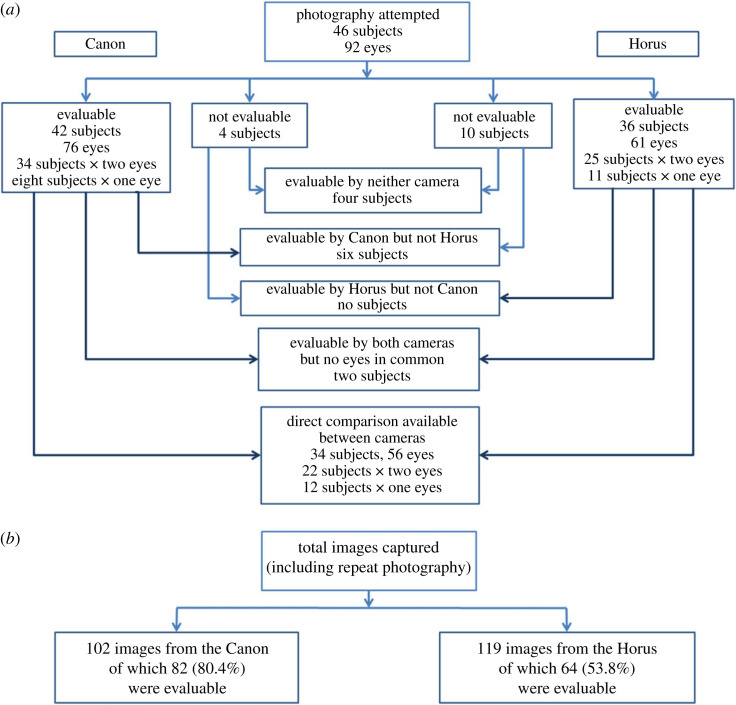
(*a*) The results of the qualitative assessment of evaluability of images from the desktop Canon and handheld Horus retinal cameras. (*b*) Summary of the total images captured.

The principal results from the qualitative visual assessment of the Horus images compared with the Canon images are shown in figure 4:

During initial trials of the Horus camera with human subjects, greater difficulty was found in acquiring adequate images compared with a desktop non-mydriatic retinal camera. From the total of 46 individuals, at least one evaluable image was obtained in 42 subjects (91%) using the Canon and 36 subjects (78%) using the Horus (*p* = 0.03). For all images captured, including repeats, more images were judged to be evaluable using the Canon (80%) than with the Horus (54%) (*p* < 0.001). In total, there were 56 image pairs of the same eye obtained with both cameras that were judged to be evaluable; these were used for further comparative quantitative analysis.

### 3.3. Results from blood vessel segmentation and metrics assessments

Following the criteria described in §2.3, table 2 shows the distribution of images under- or over-segmented by type of camera. There was evidence of a difference in the proportions of under- and over-segmentation by camera type using a Fisher’s exact test (*p* < 0.001).

**Table 2.** Distribution of under- and over-segmented images by camera type from a total of 56 common image pairs.

image source	under-segmented	over-segmented	total
Horus only	11	8	19
Canon only	1	0	1
both	1	2	3
total	13	10	23

**Figure 5.**
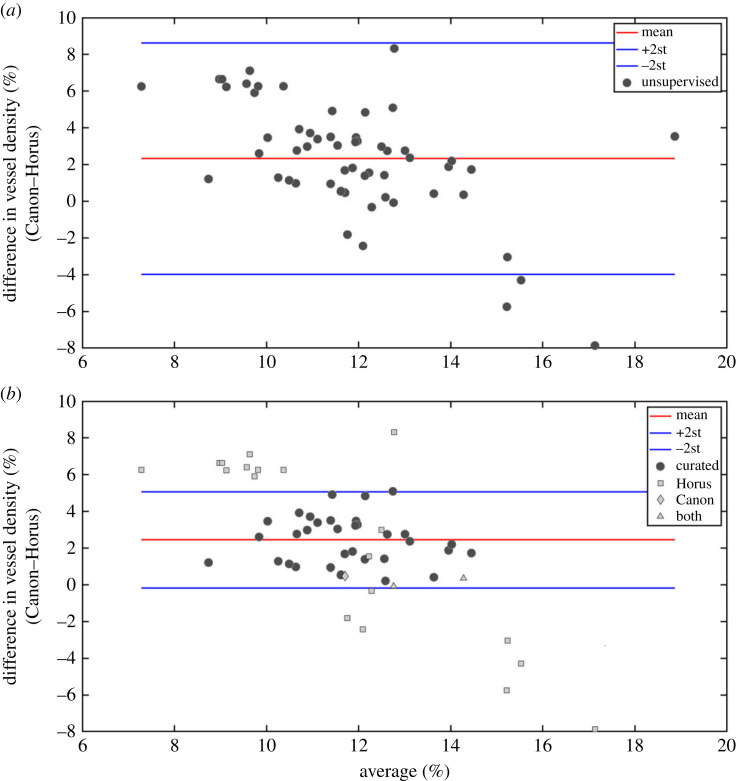
(*a*) Difference in vessel density (%) (Canon–Horus) in the unsupervised images (*n* = 56). Red line corresponds to the mean difference, and blue lines correspond to the limits of agreement (2.32 ± 3.15). (*b*) Difference in vessel density (%) (Canon–Horus) in the curated images (*n* = 33). The results for the rejected images are shown in light grey: squares are under- or over-segmentations observed in Horus (*n* = 19), diamonds observed in Canon (*n* = 1) and triangles observed in both (*n* = 3) (2.45 ± 1.31).

**Figure 6.**
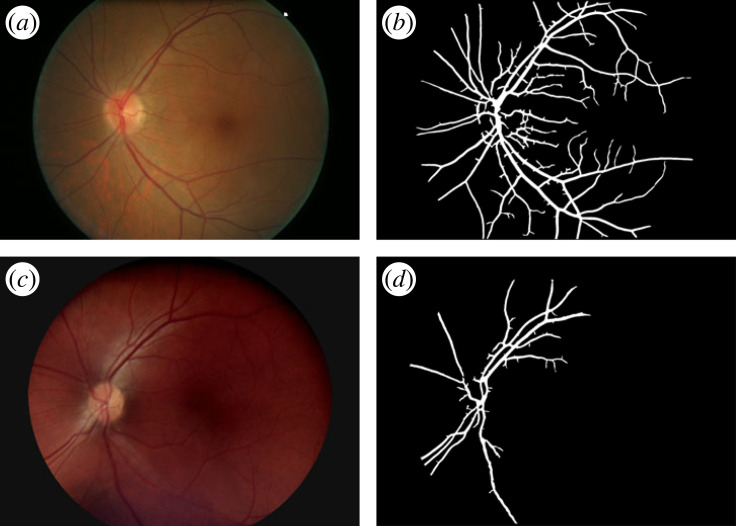
Under-segmentation. (*a*) Original Canon image, (*b*) segmented Canon, (*c*) original Horus image and (*d*) segmented Horus.

**Figure 7.**
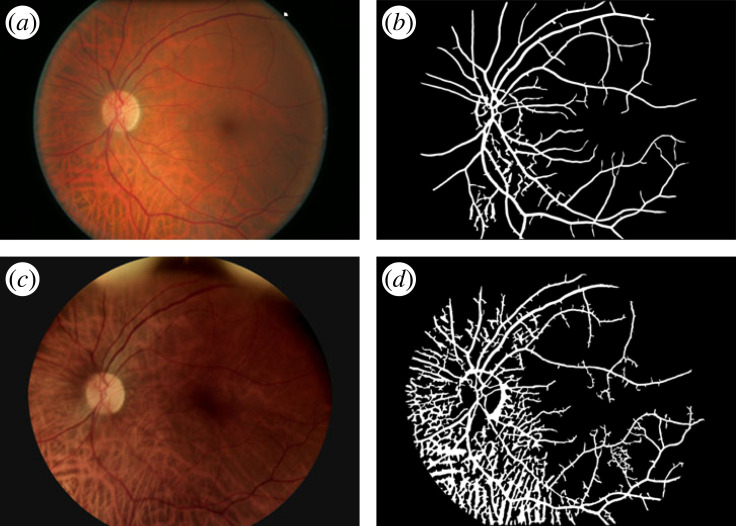
Over-segmentation. (*a*) Original Canon image, (*b*) segmented Canon, (*c*) original Horus image and (*d*) segmented Horus.

In relation to the four metrics tested, the results are as follow:

1. *Vessel density*. The vessel average density (%) for the unsupervised images was 13.05±1.71% (mean ± s.d.) for Canon, and 10.73 ± 3.29% for Horus. The Bland & Altman plot of vessel density differences (Canon–Horus) showed a bias between the Canon and Horus measures: Horus measures were on average 2.32% lower than Canon (BB test *p* < 0.001) and the limits of agreement were 2.32 ± 3.15. There was also evidence of a negative correlation between the difference and the mean (slope test *p* < 0.001), such that the extent of underestimation by the Horus was greater for higher mean values of vessel density (figure 5*a*).
  Given these results, we made a closer inspection of the data to find out the source of the bias. Following the information described in table 2, we looked at those cases which represent either Horus or Canon being under- or over-segmented. Figure 6*b* (Canon) and 6*d* (Horus), shows one example: the Horus image in figure 6*c* is so dark and has such poor contrast that the segmentation algorithm failed to identify vessels in at least three-quarters of the image (figure 6*d*), whereas figure 7*d* (Horus) shows an example with a significant number of false positives (elongated white areas on the background that are not retinal vessels), compared with that of Canon in figure 7*b*.
  Figure 5*b* shows the same data as figure 5*a*, but this time we marked with light grey figures those pairs that were judged to be either under-segmented or over-segmented. Squares are those where under/over segmentation was observed in Horus images (*n* = 19), diamonds are those where it was observed in Canon images (*n* = 1) and triangles are where it was observed in both (*n* = 3). If we just consider the curated image pairs, marked in dark grey in figure 5*b* (*n* = 33), the average percentage of vessel density for Canon was 12.98±1.40%, whereas for Horus it was 10.53±1.48%. In this case, the average difference (Canon–Horus) of vessel density percentage between Canon and Horus, was of 2.44 ± 0.13 (*n* = 33, *p* < 0.001, slope = 0.69).
2. *Histogram of length of edges*. Histograms for edges (vessels) in Canon and Horus images showed a skewed distribution, with shorter vessels having a much higher frequency than long vessels. In this case, the comparison between normalized distributions was made using the Kolmogorov–Smirnov test to compare the distributions. There was no evidence of a difference in distributions *p* = 0.95 for unsupervised images (*n* = 56) and for curated images (*n* = 33) *p* = 0.60.
3. *Number of nodes per degree and number of edges per image*. External nodes (EN) = terminal points, internal nodes (IN) = bifurcation and crossing points. For each of these graph metrics (EN, IN and number of edges), the differences (Canon–Horus) between Canon and Horus were computed for unsupervised images (*n* = 56) and for curated images (*n* = 33). For most metrics, there was a clear bias, with Horus underestimating metrics in comparison with Canon. Results are summarized in table 3.
4. *FD*. Using the skeleton lines of the segmented vessels, the FD was calculated for each image and the differences (Canon–Horus) between Canon and Horus was computed for unsupervised (*n* = 56) and curated images (*n* = 33). In both cases, Horus gave lower estimates of FD than Canon; results are summarized in table 3.

**Table 3.** Evaluation of the different metrics. Bland & Altman analysis of the differences (Canon–Horus) of the metrics between images. *p* joint and *p* slope values correspond to the respective Bradley–Blackwood test. (*a*) Upper table for unsupervised image (*n* = 56) and (*b*) lower table for curated image (*n* = 33) pairs. Italic *p*-values correspond to a failure to reject the null hypothesis. LOA (limits of agreement).

metric	mean difference (LOA)	*p* joint	*p* slope
(*a*) unsupervised image pairs (*n* = 56)			
vessel density	2.323 ± 3.151	<0.001	<0.001
external nodes	−19.982 ± 46.543	<0.001	<0.001
internal nodes	−18.589 ± 76.312	<0.001	<0.001
edges	−35.018 ± 136.110	<0.001	<0.001
fractal dimension	0.035 ± 0.042	<0.001	<0.001
(*b*) curated image pairs (*n* = 33)			
vessel density	2.446 ± 1.307	<0.001	*0.694*
external nodes	−13.818 ± 20.077	<0.001	0.008
internal nodes	−2.758 ± 26.638	*0.253*	*0.124*
edges	−7.364 ± 47.977	*0.103*	*0.179*
fractal dimension	0.033 ± 0.018	<0.001	*0.302*

The graphs of the rest of the tested metrics can be found in the electronic supplementary material, complementing the results of table 3.

## 4. Discussion

The main objective of this study was to evaluate the feasibility of extracting quantitative retinal microvascular measures from images captured using a portable handheld camera. Results were compared with a Canon CR-DGI retinal desktop camera (the reference device) in volunteers without pharmacological mydriasis. Normally, the selection of the portable camera would be made in an unreported preliminary study of the devices available at the start of the study. However, our experience in selecting a portable camera may be of interest to readers wishing to use a cheap, portable camera in their studies and so we have included a brief discussion of how we chose the Horus DEC 200 for our study in §§2.1 and 3.1.

Images from both the Horus and Canon cameras were initially subjected to a qualitative assessment, which indicated that greater difficulty was experienced in achieving evaluable images from the Horus camera than for the Canon: 36 subjects, 61 eyes and 64 images evaluable from the Horus, compared with 42 subjects, 76 eyes and 82 images from the Canon (figure 4).

From images judged qualitatively to be evaluable by the initial visual assessment, it was possible to identify 56 pairs of evaluable images taken from the same eye in 34 subjects using both cameras (Horus versus Canon). These image pairs were subjected to automatic segmentation of the retinal vessels and OD, followed by evaluation of the four metrics described in §2.2.

As described in §2.3, a further evaluation of the quality of segmentation was performed, which revealed that some images exhibited under-segmentation, whereas others suffered the opposite phenomenon of over-segmentation (table 2). In this regard, the Horus camera again exhibited a larger number of images suffering failure of segmentation: 19 failures in Horus images, three in both cameras, and one in the Canon images.

Since it was possible that some of the differences between the Canon and Horus camera related to the segmentation algorithm used, we explored whether other available AI-based methods performed differently. We tested SegRAVIR [27] and SA-UNet [28], but this required that we resize images since neither of these have been optimized for images generated by the Canon or Horus cameras. Qualitatively both methods were consistent with our findings using our algorithm [19], namely that Horus images were of substantially poorer quality than the reference Canon images and were likely to be unsuitable for reliable quantitative analysis. Due to the requirement to resize images, it was not possible to perform a more quantitative assessment using these methods. For future work, it might be interesting to consider training these networks for the appropriate image size required for our quantitative measurements. Figures with some illustrative results can be found in the electronic supplementary material.

After exclusion of images exhibiting failure of segmentation, the four metrics were compared over a total of 33 pairs of images (lower table 3). Vessel density in Horus images was significantly lower (by 2.45%±1.31) compared with the Canon, although there was no evidence of a proportional bias in the Bland & Altman plot (figure 5*b*) after removal of poorly segmented images. Similarly, significant differences (Canon–Horus) between the Horus and Canon were found in two other metrics, the FD and EN, but not in IN or edges (lower table 3).

There are several contributory factors that may be implicated in these differences in performance:

— Inevitably with a portable handheld camera, the positioning of the camera relative to the subject is less stable. In a desktop camera, the subject’s head is stabilized by a chin rest and forehead strap, thus maintaining the position of the eye relative to the optical axis of the camera. It is tempting to speculate whether the performance of a handheld camera such as the Horus might be improved by development of a simple table-top stand that could support the subject’s chin while also offering a cradle to stabilize the position of the camera. Such a device could be sufficiently portable to be carried along with the camera.
— Generally, in desktop retinal cameras (including the Canon used in this study) a number of tools are provided to aid the photographer in achieving the optimum position of the camera relative to the subject’s eye. Such tools indicate clearly and precisely what movement of the camera, both laterally and vertically is required to achieve an optimal image. While a simple feature is provided in the Horus (a dot should ideally be positioned in the middle of a square box) the photographers found this to be less intuitive and more difficult to use than the tools available on the Canon. Further development of such tools in handheld cameras to aid the photographer in camera positioning might lead to improvement in image quality.
— A particular feature of the Horus camera is that once capture of an image has been initiated, an autofocus operation is performed, introducing a delay before firing the white light flash to capture the image. During this delay, the composition screen presented to the photographer becomes blank, and feedback to the photographer on camera positioning and image composition is lost. Movement of the camera and/or subject may occur during this interval without being recognized. A pre-focus operation prior to composition of the image could lead to a worthwhile reduction in the time between initiating image capture and firing the flash, reducing the likelihood of relative movement confounding the image quality.
— Several Horus images (11 of 56, compared with 2 of 56 for Canon) appeared to suffer from underexposure and poor contrast between vessels and background, leading to under-segmentation. Given the small screen presented to the photographer on a handheld camera, it is difficult to assess the quality of the image and particularly to visualize the vascular trees in detail. This difficulty is not present in a desktop camera, where the resulting image can be seen on a large monitor and studied in detail. Photographers are reluctant to impose additional discomfort by repeated photography and so they tend to accept an image unless there is a clear defect; such defects may not be sufficiently obvious on a very small screen. To avoid this, there would be value in incorporating a feature into handheld cameras that would evaluate the exposure of the image once captured and prompt the photographer to make an appropriate adjustment and capture a further image if necessary.
— Some Horus images (8 of 56, compared with 2 of 56 for Canon) appeared to suffer from the over-segmentation phenomenon mentioned earlier. The reason why the Horus camera appeared notably more susceptible to this phenomenon compared with the Canon is unclear. We speculate that it may conceivably arise from some image processing (of which we are unaware) performed in the Horus camera that might accentuate artefacts from choroidal vessels and/or retinal nerve fibres.
— The Horus produces heavily compressed jpeg images, whereas the Canon images were captured in uncompressed tiff format. In previous unpublished work by our group, it was found that jpeg compression compromised measurements of vessels in retinal images, and accordingly capture of uncompressed or lightly compressed images is preferred for the purpose of vascular measurements. The ability to configure the level of compression applied to captured images would be a valuable addition to handheld devices such as the Horus.

We suggest that following attention to the above issues, it is conceivable that handheld retinal imaging devices such as the Horus might be capable of yielding images suitable for making microvascular measurements, subject to further evaluation. We are also aware that handheld retinal cameras are evolving rapidly and that, since this study was performed, further portable retinal imaging devices have appeared on the market. These may improve on the performance reported here.

## 5. Conclusion

In conclusion, the Horus camera was found to be the only suitable available handheld device among those tested in §3.1; however, the quantitative analysis made in §3.3 revealed a number of shortcomings compared with the Canon desktop camera. Images from the Horus camera were more likely to suffer defects compromising evaluability and/or segmentation, and also exhibited lower vessel density, even after exclusion of images exhibiting obvious defects. As always, a prospective user will have to decide if the advantages of portability and cost outweigh the disadvantages of poorer image quality. We hope that our quantitative comparisons will aid in this decision.

A number of issues have been identified which might account for these differences in performance, and measures proposed to mitigate their effect. Attention to such measures might lead to improvements in performance, allowing images from portable retinal cameras such as the Horus to be used for quantitative retinal microvascular measurements in the field.

## Data Availability

The electronic supplementary material and the Matlab code for retinal blood vessel segmentation are available on FigShare [29].

## References

[1] Wolfensberger TJ, Hamilton AM. 2001 Diabetic retinopathy – an historical review. Semin. Ophthalmol. **16**, 2-7. (10.1076/soph.16.1.2.4220) 15487691

[2] Wong TY, McIntosh R. 2005 Systemic associations of retinal microvascular signs: a review of recent population-based studies. Ophthalmic Physiol. Opt. **25**, 195-204. (10.1111/j.1475-1313.2005.00288.x) 15854064

[3] Wong TY. 2014 Improving the prediction of hypertensive target organ damage using novel markers: lessons from retinal vascular imaging research. Hypertension **64**, 233-234. (10.1161/HYPERTENSIONAHA.114.03479) 24866138

[4] Witt N, Wong TY, Hughes AD, Chaturvedi N, Klein BE, Evans R, McNamara M, Thom SM, Klein R. 2006 Abnormalities of retinal microvascular structure and risk of mortality from ischemic heart disease and stroke. Hypertension **47**, 975-981. (10.1161/01.HYP.0000216717.72048.6c) 16585415

[5] Tapp RJ, Williams C, Witt N, Chaturvedi N, Evans R, Thom SAM, Hughes AD, Ness A. 2007 Impact of size at birth on the microvasculature: the Avon Longitudinal Study of Parents and Children. Pediatrics **120**, e1225-e1228. (10.1542/peds.2006-2951) 17974715 PMC2780696

[6] Tapp RJ et al. 2015 Impact of blood pressure on retinal microvasculature architecture across the lifespan: the Young Finns Study. Microcirculation **22**, 146-155. (10.1111/micc.12187) 25559612

[7] Karakaya M, Hacisoftaoglu RE. 2020 Comparison of smartphone-based retinal imaging systems for diabetic retinopathy detection using deep learning. BMC Bioinf. **21**, 259. (10.1186/s12859-020-03587-2) PMC7336606 32631221

[8] Palermo BJ, D’Amico SL, Kim BY, Brady CJ. 2022 Sensitivity and specificity of handheld fundus cameras for eye disease: a systematic review and pooled analysis. Surv. Ophthalmol. **67**, 1531-1539. (10.1016/j.survophthal.2021.11.006) 34822849

[9] Nunez do Rio JM, Nderitu P, Raman R, Rajalakshmi R, Kim R, Rani PK, Sivaprasad S, Bergeles C. 2023 Using deep learning to detect diabetic retinopathy on handheld non-mydriatic retinal images acquired by field workers in community settings. Sci. Rep. **13**, 1392. (10.1038/s41598-023-28347-z) 36697482 PMC9876892

[10] EyeCare. 2015 D-EYE. See http://www.d-eyecare.com (accessed June 2019).

[11] Peek Vision. 2017 Peek Retina. See http://www.peekvision.org/ (accessed March 2019).

[12] WelchAlleyn. 2013 PanOptic + iExaminer. See http://www.welchallyn.com/en/microsites/iexaminer.html (accessed April 2019).

[13] Volk. 2016 Volk iNview. See https://www.volk.com/products/inview-for-iphone-6-6s (accessed March 2019).

[14] MiiSHsinchu. 2017 Horus DEC 200. See http://www.miis.com.tw/index.php?lang=us (accessed June 2019).

[15] Edmund Optics. 2011 USAF chart. See https://www.edmundoptics.com/f/pocket-usaf-optical-test-pattern/12147/">www.edmundoptics.com/f/pocket-usaf-optical-test-pattern/12147/ (accessed May 2018).

[16] Canon. 2003 CR-DGi fundus camera. See https://us.medical.canon/products/eye-care/.

[17] Chapman N, Witt N, Gao X, Bharath AA, Stanton AV, Thom SAM. 2001 Computer algorithms for the automated measurement of retinal arteriolar diameters. Br. J. Ophthalmol. **85**, 74-79. (10.1136/bjo.85.1.74) 11133716 PMC1723694

[18] Zuiderveld K. 1994 Contrast limited adaptive histogram equalization. In *Graphics gems IV* (ed. PS Heckbert), pp. 474–485. New York, NY: Academic Press.

[19] Martinez-Perez ME, Hughes AD, Thom SAM, Bharath AA, Parker KH. 2007 Segmentation of blood vessels from red-free and fluorescein retinal images. Med. Image Anal. **11**, 47-61. (10.1016/j.media.2006.11.004) 17204445

[20] Martinez-Perez ME, Witt N, Parker KH, Hughes AD, Thom SAM. 2019 Automatic optic disc detection in colour fundus images by means of multispectral analysis and information content. PeerJ **7**, e7119. (10.7717/peerj.7119) 31293825 PMC6599671

[21] Lam L, Lee SW, Suen CY. 1992 Thinning methodologies—a comprehensive survey. IEEE Trans. Pattern Anal. Mach. Intell. **14**, 869-885. (10.1109/34.161346)

[22] Mandelbrot BB. 1983 The fractal geometry of nature. New York, NY: W. H. Freeman.

[23] Bland JM, Altman DG. 1986 Statistical methods for assessing agreement between two methods of clinical measurements. Lancet **1**, 307-310. (10.1016/S0140-6736(86)90837-8) 2868172

[24] Bradley EL, Blackwood LG. 1989 Comparing paired data: a simultaneous test for means and variances. Am. Stat. **43**, 234-235. (10.1080/00031305.1989.10475665)

[25] Hayes K, OBrien K, Kinsella A. 2017 A decomposition of the Bradley-Blackwood paired-samples omnibus test. Commun. Stat. Theory Methods **46**, 9892-9896. (10.1080/03610926.2016.1222439)

[26] Wang S, Jin K, Lu H, Cheng C, Ye J, Qian D. 2016 Human visual system-based fundus image quality assessment of portable fundus camera photographs. IEEE Trans. Med. Imaging **35**, 1046-1055. (10.1109/TMI.2015.2506902) 26672033

[27] Hatamizadeh A, Hosseini H, Patel N, Choi J, Pole CC, Hoeferlin CM, Schwartz SD, Terzopoulos D. 2022 RAVIR: A dataset and methodology for the semantic segmentation and quantitative analysis of retinal arteries and veins in infrared reflectance imaging. IEEE J. Biomed. Health Inform. **26**, 3272-3283. (10.1109/JBHI.2022.3163352) 35349464

[28] Guo C, Szemenyei M, Yi Y, Wang W, Chen B, Fan C. 2021 SA-UNet: spatial attention U-Net for retinal vessel segmentation. In *2020 25th Int. Conf. on Pattern Recognition (ICPR), Milan, Italy*, pp. 1236–1242. (10.1109/ICPR48806.2021.9413346)

[29] Martinez-Perez ME, Hughes AD, Thom SAM, Parker KH, Witt NH. 2023 Supplementary material for Evaluation of a portable retinal imaging device: towards a comparative quantitative analysis for morphological measurements of retinal blood vessels. Figshare. (10.6084/m9.figshare.c.6693830)

